# Influence of Gamma irradiation on shape memory polymer nano-composite for satellite deployment mechanism

**DOI:** 10.1038/s41598-024-73676-2

**Published:** 2024-10-13

**Authors:** Emad Mousa, Eman O. Taha, Salah Lotfy, Ahmad Anwar

**Affiliations:** 1 Physics Department, Faculty of Science, Cairo University, Giza, 12613 Egypt; 2 Petroleum Applications Department, Egyptian Petroleum Research Institute (EPRI), Cairo, Egypt; 3 Polymer Chemistry Department, National Center for Radiation Research and Technology (NCRRT), Egyptian Atomic Energy Authority (EAEA), Cairo, Egypt; 4 Mechanical Engineering Department, Faculty of Engineering, Pharos University in Alexandria (PUA), Alexandria, Egypt

**Keywords:** Shape memory polymers, Nano-composite, Gamma irradiation, Satellite deployment, Materials science, Physics

## Abstract

This research investigates the impact of gamma irradiation on epoxy-MWCNT nanocomposites for satellite deployment mechanisms. Nanocomposites, enhanced with surfactants, were meticulously prepared and subjected to controlled gamma irradiation (250–1000 kGy) utilizing the Cobalt-60 facility Industrial Mega Gamma-1 at NCRRT in Egypt. Surface tension measurements explored surfactant effects on epoxy-MWCNT composites in acetone. Acetone reduced tension from 26.7 to be 24.2 (mN/m). Surfactants (Tween 80, SDS) effectively lowered tension (24.4 mN/m), while surfactant-free systems had higher tension (25.1 mN/m). Cationic surfactant (CTAB) slightly increased tension (25.4 mN/m) but aided MWCNT dispersion. Nonionic and anionic surfactants showed superior dispersing power, aligning with MWCNTs and enhancing dispersion. Thermogravimetric analysis (TGA) unveiled alterations in the thermal stability of epoxy-MWCNT nanocomposites induced by radiation, particularly evident at elevated doses (500 and 1000 kGy). Notably, surfactant-modified specimens exhibited discernible effects on various thermal stability parameters. DMA analysis revealed radiation-induced changes in viscoelastic properties. Unirradiated epoxy exhibited a T_g_ of 58 °C, while 250 kGy irradiation enhanced crosslinking (T_g_: 64 °C). Higher doses (500 kGy, 1000 kGy) caused marginal T_g_ changes. Surfactant-modified samples showed varied effects, with Tween 80 emphasizing its role in phase separation. Results highlighted radiation’s influence on stiffness and energy dissipation. Shape memory behavior indicated increased recovery time with higher doses, except at 250 kGy. Epoxy-MWCNT exhibited a stable recovery time, suggesting a MWCNT stabilizing effect. Fixation rates consistently reached 100%, indicating improved shape recovery influenced by MWCNTs and surfactants. This study provides insights into optimizing nanocomposites for satellite deployment applications.

## Introduction

In the challenging environment of space, the demand for advanced, durable, and lightweight materials is crucial. Spacecrafts are exposed to a range of severe environmental conditions, including vacuum, hypervelocity impacts by micrometeoroids and debris, extreme thermal cycling, Vacuum ultraviolet (VUV) and ionizing radiation. Additional threats, such as atomic oxygen (AO), are encountered in low Earth orbit (LEO) altitudes^1–4^. Herein, spacecraft structures require high-performance materials capable of withstanding the demanding conditions of space.

Shape memory polymers (SMPs) are a class of materials that can be triggered to return to their pre-deformed shape through external stimuli like light, heat, electric or magnetic fields, pH level, or ionic strength^5–9^. Recently, SMPs have emerged as exceptional materials for spacecraft applications, especially deployable structures, due to their unique properties such as low density, extensive recoverable strain, and extended recovery time^10,11^. These characteristics make SMPs ideal substitutes for heavy metal-based mechanisms in spacecrafts. Indeed, thermoset SMPs are particularly attractive for space structures, as they are characterized by high material stiffness, high glass transition temperature, and excellent environmental durability^12,13^.

Epoxy-based SMPs are highly attractive for space applications due to their low outgassing properties, high triggering temperature, and high strength-to-weight ratio^14^, in addition to excellent shape recovery ratios and elastic moduli. Epoxy can be reinforced with carbon fibers (CFs) to create composites for various applications in space structures, such as hinges, solar arrays, deployable panels, booms, and reflector-antennas^15^. Moreover, the addition of graphite, carbon particles, or fibers can significantly enhance the strength and stiffness of epoxy^16^. Notably, epoxy nanocomposites reinforced with multi-walled carbon nanotubes (MWCNTs) have demonstrated the ability to endure space radiation exposure of up to 1 MGy without significant degradation in their mechanical properties^17–19^. However, the interface between MWCNTs and epoxy matrix is one of the key challenges, as it plays a crucial role in optimizing the properties and performance of the nanocomposite^20^.

When exposed to ionizing radiation, such as gamma rays, shape memory polymers and nanocomposites can undergo various changes in their properties^21^. These changes include modifications in mechanical, thermal, and shape memory properties, as well as changes in the molecular structure and crosslinking density of the materials^22,23^. Understanding these effects is crucial for ensuring the reliability and performance of spacecraft structures.

In short, the aim of the present research is to investigate the influence of gamma irradiation, as a simulation of electromagnetic radiation affecting spacecraft structures, on epoxy/MWCNT nanocomposites as shape memory materials for spacecraft structures. The radiation dose and the type of surfactant used to disperse MWCNTs in epoxy are two key factors in this study.

## Materials and methods

### Materials

The multi-walled carbon nanotubes (MWCNTs), varying in length from 4 to 10 μm, with an average outer diameter of 8 to 10 nm, an inner diameter of 4 nm, and possessing up to 15 walls, were obtained from the Egyptian Petroleum Research Institute (EPRI) and synthesized using the chemical vapor deposition method (CVD), the MWCNTs demonstrate a purity level surpassing 90%. The epoxy resin system employed, Biresin^®^ CR82, consists of two components: Biresin CR-82 part A and CH-80 6-part B (hardener), obtained from Sika Advanced Resins (SIKA Deutschland GmbH), Germany. This epoxy resin system has been previously employed in various published studies^24–26^. The surfactants, including Tween 80, SDS (> 99%), and CTAB (99%), were sourced from various suppliers: MP Biomedicals, Inc., El Nasr Pharmaceutical Chemicals Co (ADWIC), and Sigma Aldrich Chemical Co., respectively. All chemicals were used without additional purification.

## Preparation of the epoxy-MWCNTs nanocomposites

In the preparation of the epoxy-MWCNTs nanocomposites, enhancing the dispersion of MWCNTs within the epoxy resin was achieved through the utilization of three types of surfactants: a nonionic (neutral) surfactant, Tween 80; an anionic surfactant, SDS; and a cationic surfactant, CTAB. A relatively elevated concentration of each surfactant, equivalent to 15 times the critical micelle concentration (CMC), was dissolved in the organic solvent acetone. Surpassing the CMC threshold results in the formation of micelles, thereby facilitating improved dispersion. The specific quantities of surfactants utilized are detailed below:

CTAB: Approximately 0.55 g in 100 ml for 15CMC.

Tween 80: Approximately 0.021 g in 100 ml for 15CMC.

SDS: Approximately 0.23 g in 100 ml for 15CMC.

**Fig. 1:**
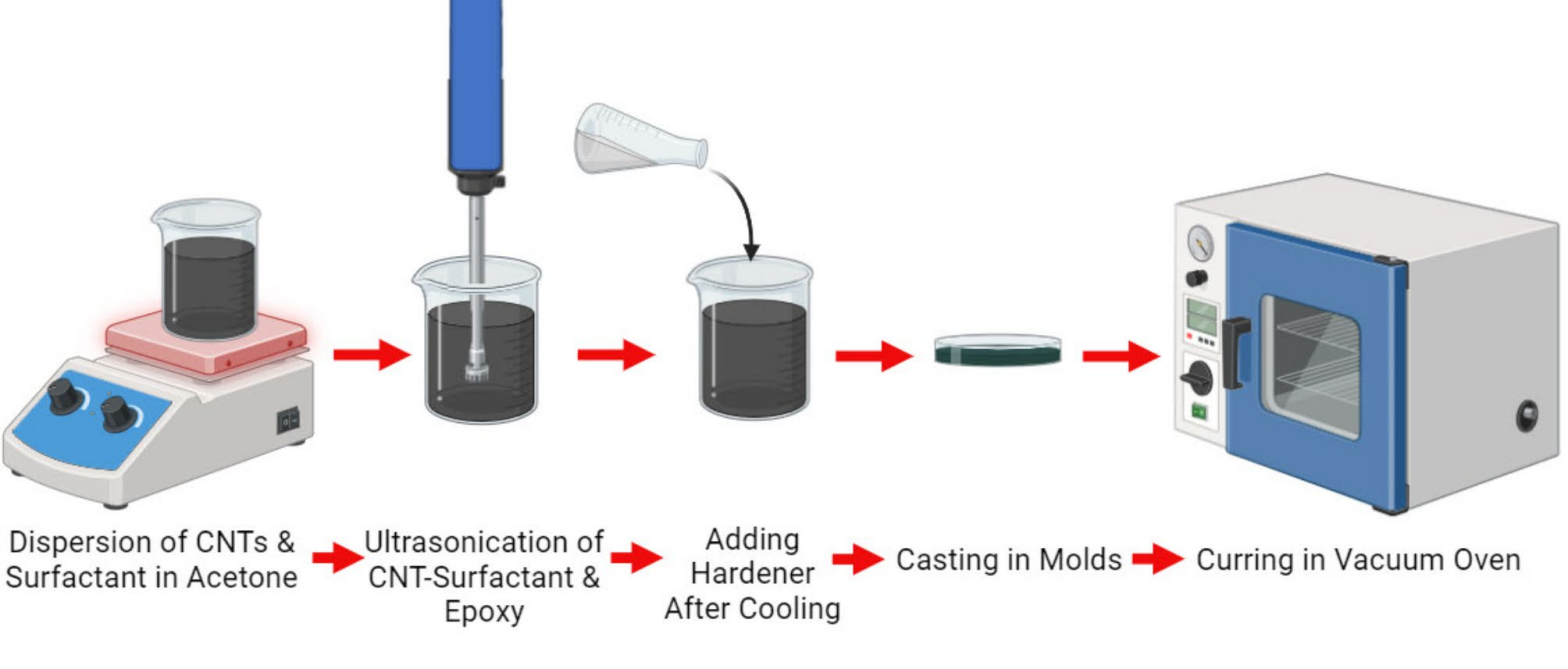
Schematic representation of fabrication process for the epoxy-MWCNTs nanocomposites.

The whole fabrication process is represented schematically in Fig. 1. However, the procedure outlined by Kim et al.^27^ involved dispersing MWCNTs (0.5% by weight of the polymer matrix) and their respective surfactant (15 CMC) in acetone. Ultra-sonication was conducted using a 6 mm horn of an ultrasonic homogenizer (model TU-65-Y) at 650 W power, with a 40% power amplitude, applying a 5-second pulse on and a 1-second pulse off, for a total duration of 60 min to ensure effective dispersion. After cooling to room temperature, the hardener (CH-80-6-part B) was added to initiate curing of the epoxy resin system. The resulting MWCNTs-epoxy nanocomposite mixture was cured in both aluminum molds and silicone pans. Subsequently, vacuum treatment (< 200 mbar) at 50 °C for 24 h was carried out to eliminate the organic solvent and any trapped bubbles. The compositions of the prepared epoxy-MWCNTs nanocomposites are presented in Table 1.

**Table 1:** Composition of the prepared epoxy-MWCNTs nanocomposites.

Sample number	Epoxy (weight%)	CNT (weight%)	Surfactant type	Surfactant concentration (g in 100 ml)
1	100	0	-	
2	99.5	0.5	-	
3	99.5	0.5	SDS (anionic)	0.23
4	99.5	0.5	CTAB (cationic)	0.55
5	99.5	0.5	Tween 80 (neutral)	0.021

## Gamma irradiation of epoxy nanocomposite material using Cobalt-60 source

Slides of epoxy nanocomposites were exposed to gamma irradiation using the Cobalt-60 facility named Industrial Mega Gamma-1, specifically the IR-206 (JS-9500) NCRRT model. This facility is manufactured by MDS-Nordion Inc., a Canadian company. The facility is located at the Egyptian Atomic Energy Authority’s National Centre for Radiation Research and Technology (NCRRT) in Egypt. During the irradiation process, the epoxy nanocomposite specimens were placed in the facility under controlled conditions, including ambient air, moisture, and room temperature. The specimens were exposed to radiation doses of 250, 500, and 1000 kGy. The dose rate used for the irradiation process was consistent at 0.72 kGy/h.

## Surface tension measurements

Using a Force Tensiometer K20, Version 1.25, the surface tension measurements were conducted. The ring method was applied with specific settings: the stage speed was adjusted to 25%, the reading limit was set to 10, with a standard deviation 5 mN/m, and an acceleration due to gravity of 9.807 m/s². Correction based on empirically derived tables by Harkins and Jordan (H&J)^28^ were implemented.

The surface tension (σ) was determined using the formula:

$$\sigma=\frac{F}{L\,{\star}\cos\theta}$$

​In this equation, F denotes the maximum force (Fmax), while L refers to the wetted length of the ring, which includes both the inner and outer circumferences.

## Thermogravimetric analysis (TGA)

TGA was used for measuring the thermal decomposition of the prepared samples at a rate of 10 °C / minute using a Mettler Toledo TA-TGA system from room temperature to 700 °C.

### Dynamic mechanical analysis (DMA)

Dynamic mechanical analyses of the samples were investigated by DMA analyzer (MetraviB + 25 Instrument, France) in strain mode at fixed frequency of 1 Hz. Samples were heated from 35 °C to 100 °C at a heating rate of 2 °C / min.

## Shape memory behavior analysis

To examine the influence of gamma radiation on epoxy-based nanocomposites as shape memory materials for spacecraft structures, a comprehensive study was undertaken. The shape memory behavior of the produced epoxy nanocomposites was evaluated through a bending test, following the experimental methodology outlined by Lendlein and Kelch^29,30^. In the bending test, the epoxy nanocomposite material slide samples were subjected to an initial twist at a temperature above the glass transition point (T_g_) of the material, resulting in an angle denoted as θ_0_. Subsequently, the sample was cooled to 0 °C while maintaining the deformation by applying an external force (θ_i_). The twisted sample was then immersed in water at a temperature higher than T_g_, facilitating the recovery of its permanent shape. Throughout the recovery process, the variation in angle (θ_f_) over time was carefully monitored and recorded.

To quantify the shape memory behavior, two essential ratios were defined. The recovery ratio (R_r_) was determined by calculating (θ_i_ - θ_f_) / θ_i_, providing a measure of the sample’s capability to regain its original shape. Additionally, the fixity ratio (R_f_) was defined as θ_i_ / θ_0_, enabling the assessment of the degree of deformation in the initial twisted configuration. The main aim of this experimental methodology was to assess the shape memory characteristics of the epoxy nanocomposites and explore the impact of gamma irradiation on their shape memory performance. Such insights are crucial for exploring the potential utilization of these materials in spacecraft structures.

## Results and discussion

### Surface tension

Surface tension measurements were performed to examine the impact of surfactants on epoxy resin composites containing MWCNTs distributed in acetone. To provide a clear comparison of the surface tension values for the different systems tested, surface tension data are summarized in Table 2. The findings offer important insights into the dispersing capabilities of various surfactants and their interactions with the composite components:

Impact of Acetone: Introducing acetone into the epoxy resin slightly reduced the surface tension from 26.7 to be 24.2 (mN/m). This reduction indicates that acetone, a commonly used solvent, decreases surface tension by disrupting intermolecular forces^31^.

Surfactant-Free System: In the absence of a surfactant, the epoxy-MWCNTs acetone composite exhibited a slightly higher surface tension of 25.1 mN/m. This indicates a lower dispersing power, potentially leading to less effective distribution of MWCNTs throughout the epoxy resin.

Nonionic and Anionic Surfactants: Tween 80 and SDS surfactants both effectively reduced the surface tension of the epoxy MWCNT mixtures in acetone to 24.4 mN/m. This suggests that both surfactants have similar dispersing efficiencies^32,33^.

Cationic Surfactant (CTAB): The surface tension for the cationic surfactant (CTAB) was measured at 25.4 mN/m, slightly higher than that for the nonionic and anionic surfactants. Although CTAB’s dispersing power is less than that of Tween 80 and SDS, it still helps reduce surface tension and aids in the distribution of MWCNTs throughout the epoxy resin.

**Table 2:** Surface tension values for different systems.

System composition	Surface Tension (mN/m)
Epoxy-Acetone	24.2
Epoxy-MWCNTs-Acetone	25.1
Epoxy-MWCNTs-Acetone SDS	24.4
Epoxy-MWCNTs-Acetone CTAB	25.4
Epoxy-MWCNTs-Acetone Tween 80	24.4

Overall, these results underscore the superior dispersing power of nonionic and anionic surfactants (Tween 80 and SDS) in composites of epoxy resin and MWCNTs dispersed in acetone, compared to surfactant-free and cationic surfactant (CTAB) systems. The improved scattering is credited to the alignment of the surfactant molecules, orienting their hydrophobic tails towards the MWCNTs while positioning their hydrophilic heads towards the epoxy. This alignment decreases the interfacial tension between the epoxy and MWCNTs, preventing MWCNT aggregation. The physical adsorption of surfactants on the MWCNTs’ surfaces reduces surface tension and improves dispersion in the epoxy matrix. Additionally, micelle formation, induced by surfactant concentrations exceeding the critical micelle concentration (CMC), plays a crucial role in neutralizing van der Waals forces and preventing MWCNT aggregation. The micelles act as stabilizing agents, preventing re-agglomeration of dispersed MWCNTs.

It is important to recognize that the mechanisms of surfactant action and the optimal surfactant concentration can vary depending on experimental conditions, surfactant characteristics, and the specific MWCNTs and epoxy resin used^34^. Further research and characterization are needed to fully understand these interactions and optimize the dispersion of MWCNTs in epoxy composites^35^.

### Thermogravimetric analysis of (TGA)

Thermogravimetric Analysis (TGA) is a crucial method for understanding thermal degradation processes of polymers and polymer composites, and is fundamental for evaluating their thermal stability. Also, it is particularly useful for assessing the activation energy linked to thermal decomposition processes^36^. On the other hand, TGA investigations of polymer nanocomposites reveal that the kinetic parameters are influenced by several factors such as the kind of polymer, the process used for sample preparation, the chemical composition of the filler, and the concentration of the filler. Therefore, the investigation of changes in thermal characteristics caused by irradiation of polymer composites becomes a relevant study^37,38^.

**Fig. 2:**
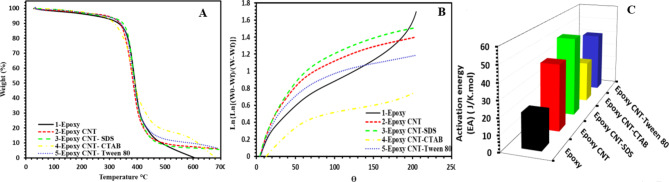
**(A)** TGA of all epoxy nanocomposites with various surfactant types, **(B)** values of ln{ln[(W_0_ - W_f_) / (W_t_ - W_f_)]} against θ, and **(C)** activation energy of all epoxy nanocomposites with various surfactant types.

Figure 2(A) displays the TGA curves of unirradiated epoxy-CNT nanocomposites with various surfactant types. Clearly, all samples display decomposition peaks between 280 °C and 450 °C, which can be related to the deterioration of the primary epoxy chains. The peak temperature, which represents the greatest rate of decomposition, differs across the samples. The values for epoxy, epoxy/CNT, epoxy/CNT-SDS, epoxy/CNT-CTAB, and epoxy/CNT-Tween 80 samples are 389.3 °C, 381.8 °C, 393 °C, 382.3 °C, and 387.7 °C, respectively. The epoxy/CNT-SDS sample has the greatest decomposition temperature (T_D_), followed by epoxy/CNT-CTAB, indicating an enhancement in thermal stability. The observed behavior is attributed to the increased crosslinking density in the MWCNTs loaded epoxy nanocomposites, as will be discussed later in (DMA) section. An increased crosslinking density in polymer enhances interchain bonding, hence improving heat resistance in nanocomposites. The epoxy/CNT-SDS sample demonstrates a decrease in the distance between crosslinking points, resulting in an increase in the crosslinking density. Moreover, the incorporation of SDS surfactant leads to the creation of a seamless network of carbon nanotubes, which in turn decreases the pace at which breakdown products evaporate. The increase in thermal stability is ascribed to the enhanced dispersion of carbon nanotubes (CNT) assisted by sodium dodecyl sulfate (SDS), aligning with findings reported in existing literature^39^.

In the examination of thermal stability, Horowitz and Metzger’s method proved instrumental for determining activation energies of thermal decomposition from TGA data. The relation employed for this purpose is expressed as^36,40,41^:

$$Ln\left[ln\left[\frac{W_o-W_f}{W-W_f}\right]\right]=\frac{E_A(\theta)}{RT_s^2}$$

Here, W_0_ and W_f_ denote the initial and final weights of specimens, W represents the specimen’s remaining weight at temperature T_s_ ​, R signifies the gas constant (8.314 JK^−1^mol^−1^), and θ = T- T_s_ (T being the specimen’s temperature). The reference temperature T_s_ ​ is defined as the temperature where.

$$\left[\frac{W-W_f}{W_o-W_f}\right]=\frac{1}{e}$$

Figure 2(B) depicts the correlation between the natural logarithm of ln [(W_0_ - W_f_) / (W - W_f_)] and θ which is used to determine the activation energy (E_A_) in thermal decomposition for all nanocomposites. The slope of the depicted straight line confirms [(E_A_ × 10^3^) / RT_s_^2^] ​, allowing for the determination of E_A_. The respective activation energy values for all studied nanocomposites are tabulated in Table 3.

The activation energy of all epoxy nanocomposites with various surfactants is displayed in Fig. 2(C). The activation energy for thermal decomposition in pure epoxy is around 21.36 kJ/mol, which is the lowest among all other samples. In contrast, epoxy/CNT sample which has been treated with sodium dodecyl sulfate (SDS), exhibits the greatest activation energy of 50.85 kJ/mol. The addition of SDS treatment results in a significant increase by 8.98 kJ/mol compared to surfactant-free epoxy/CNT sample and by 29.49 kJ/mol compared to pure epoxy sample. This suggests that SDS treatment hinders the degradation reaction. The increased crosslinking density of epoxy can be due to the even distribution of CNT-SDS, which facilitates this process.

**Table 3:** Activation energy (E_A_), and decomposition temperature (T_D_) values for all samples.

	Activation energy (E_A_) ( J/K.mol)	T_D_ (°C) 0 kGy	T_D_ (°C) 250 kGy	T_D_ (°C) 500 kGy	T_D_ (°C) 1000 kGy
Epoxy	21.36	389.3	390.6	370.5	371.1
Epoxy CNT	41.87	381.8	390.7	362.2	374.1
Epoxy CNT SDS	50.85	393	390.2	363.7	361.7
Epoxy CNT CTAB	26.92	382.3	387.9	363.3	356.2
Epoxy CNT Tween 80	40.03	387.7	391.8	365.3	363.2

**Fig. 3:**
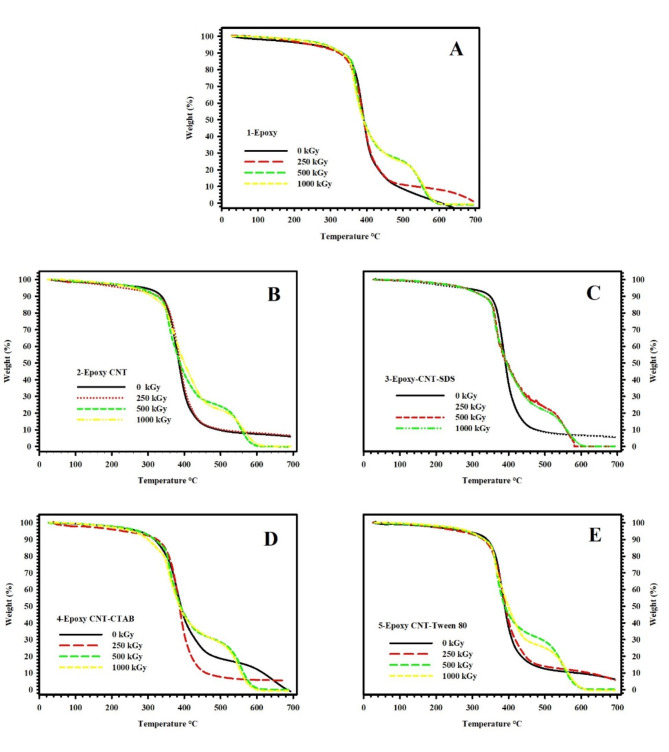
TGA of **(A)** neat epoxy, **(B)** epoxy-CNT, **(C)** Epoxy-CNT-SDS, **(D)** Epoxy-CNT-CTAB, and **(E)** Epoxy-CNT-Tween 80 nanocomposites under different gamma irradiation doses.

The impact of Gamma-radiation doses (250, 500, 1000 KGy) on the Thermogravimetric Analysis (TGA) curves of neat epoxy, epoxy-CNT, epoxy/CNT-SDS, epoxy/CNT-CTAB, and epoxy/CNT-Tween 80 nanocomposites are vividly illustrated, respectively, in Fig. 3(A), Fig. 3(B), Fig. 3(C), Fig. 3(D), and Fig. 3(E). The ensuing decomposition temperature values are methodically documented in Table 3.

Notably, the unirradiated epoxy-CNT nanocomposite and those irradiated with 250 KGy exhibit a singular step in weight loss. Conversely, the nanocomposites subjected to higher radiation doses (500 KGy and 1000 KGy) manifest a two-step weight loss process. After exposing the samples to a dose of 250 KGy of Gamma irradiation, there is a noticeable enhancement in the decomposition temperature and, consequently, the thermal stability across all samples. However, this positive trend is reversed with irradiation doses of 500 KGy and 1000 KGy, leading to a decrement in decomposition temperature and, correspondingly, a reduction in thermal stability.

The observed phenomena can be explained by the simultaneous presence of two processes during the irradiation of polymer composites: crosslinking and chain degradation. Crosslinking is a chemical reaction that stabilizes free radicals and promotes the formation of intermolecular bonds, resulting in increased thermal stability. Conversely, chain degradation, which is the second phase, occurs with greater radiation doses. This leads to the breaking down of polymeric chains into smaller units and, as a result, a decrease in thermal stability. It is crucial to recognize that the degree of crosslinking or chain degradation is significantly affected by the radiation doses in both phenomena^42–44^.

### Dynamic mechanical analysis (DMA)

DMA is a technique used to investigate and characterize materials, particularly polymers. It is most useful for investigating the viscoelastic behavior of materials, i.e., their capacity to store and release energy when subjected to stress^45^.

### Effect of radiation doses

**Fig. 4:**
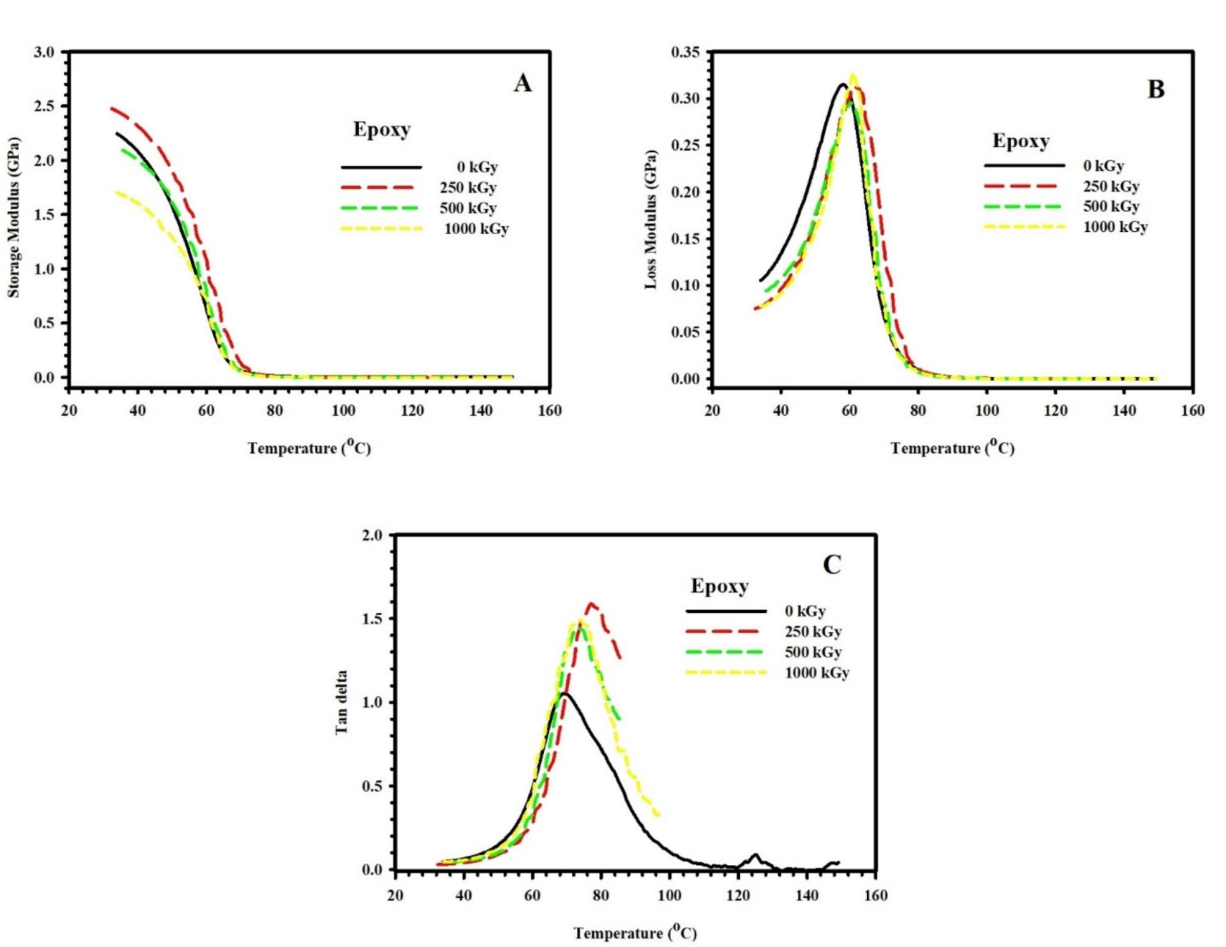
**(A)** Storage modulus (E´), **(B)** Loss modulus (E˝), and **(C)** Loss factor (tanδ) of pristine epoxy polymer, subjected to different doses of gamma radiation, as a function of temperature (35–100 °C) and fixed frequency 1 Hz.

One of the most important parameters measured by DMA is the energy storage modulus (E´), which reflects the stiffness of the material. Figure 4(A) plots the storage modulus E´ of pristine epoxy polymer, subjected to different doses of gamma radiation, as a function of temperature (35–100°C) and a fixed frequency of 1 Hz. Three regions are presented in Fig. 1a for all samples under test. Region (I) is the low-temperature glassy region ~ (T < 40°C), where E´ is almost temperature-independent. In addition, region (II) is the glass transition region (40°C < T < 70°C), where the state of the material transforms from elastic to viscous, and E´ decreases dramatically with temperature. Region (III) is the rubbery region (T > 70°C) where the material flows and exhibits viscous behavior with low E´ values. It is worth mentioning that a material with a high modulus at low temperatures will be able to store more energy in the glassy state, and thus it will be able to recover its original shape more effectively upon heating. In this case, the glassy-to-rubbery modulus ratio (E_gr_) is a key factor in specifying the shape recovery of the material^21^.

The unirradiated epoxy sample showed an E´ value of 2.25 GPa at low temperatures with the lowest E_gr_ value (0.47 × 10^3^) among all other samples. Meanwhile, epoxy sample subjected to 250 kGy dose acquire the highest value of E_gr_ (41.77 × 10^3^). Other irradiated samples reported E_gr_ values of 7.62 × 10^3^ and 0.77 × 10^3^ for higher radiation doses of 500 kGy and 1000 kGy, respectively. An alteration in E_gr_ upon irradiation can be understood on the basis of increased crosslinking of epoxy chains, which leads to an increase in E_gr_, or increased degradation of chains, which leads to a decrease in E_gr_^46^.

The loss modulus is another parameter that reflects the energy dissipation due to frictional interactions caused by the movement of polymer chains, segments, or particles. As mentioned previously, an increase in temperature leads to an increase in molecular movements. Once the timescale of molecular motion coincides with that of time-dependent mechanical deformation, the loss modulus peaks.

On the other hand, the loss factor is also employed to study the damping characteristics of a material, and it is defined as the ratio of the loss modulus to the storage modulus. Indeed, the temperature at which the loss factor reaches its maximum value is higher than the temperature at which the maximum loss modulus occurs. This is attributed to the fact that the decrease in the storage modulus in the vicinity of the glass transition region suppresses the initial increase in the loss factor^47^.

Figure 4(B) and 4(C) illustrate, respectively, the loss modulus (E˝) and loss factor (tanδ) of epoxy polymer, subjected to different doses of gamma radiation, as a function of temperature (35–100 °C) and fixed frequency 1 Hz. It is obvious from the figures that both E˝ and tanδ peak at the glass transition temperature (T_g_), as the material transforms from a glassy to a rubbery state. In this work, T_g_ will be determined using the E˝ plot^47^. T_g_ is also a key factor to specify the shape recovery of the material. SMPs with higher T_g_ value require higher energy to reach the transition temperature which may result in a slower shape recovery process.

Unirradiated epoxy sample showed the lowest value of T_g_ (58 °C). Meanwhile, epoxy sample subjected to 250 kGy dose reported the highest T_g_ value (64 °C) which confirms the chain immobilization caused by increased crosslinking rate at this radiation dose. Higher radiation doses of 500 kGy and 1000 kGy resulted in a slight change in T_g_ of epoxy samples to 60 °C and 59 °C, respectively.

### Effect of composition

**Fig. 5:**
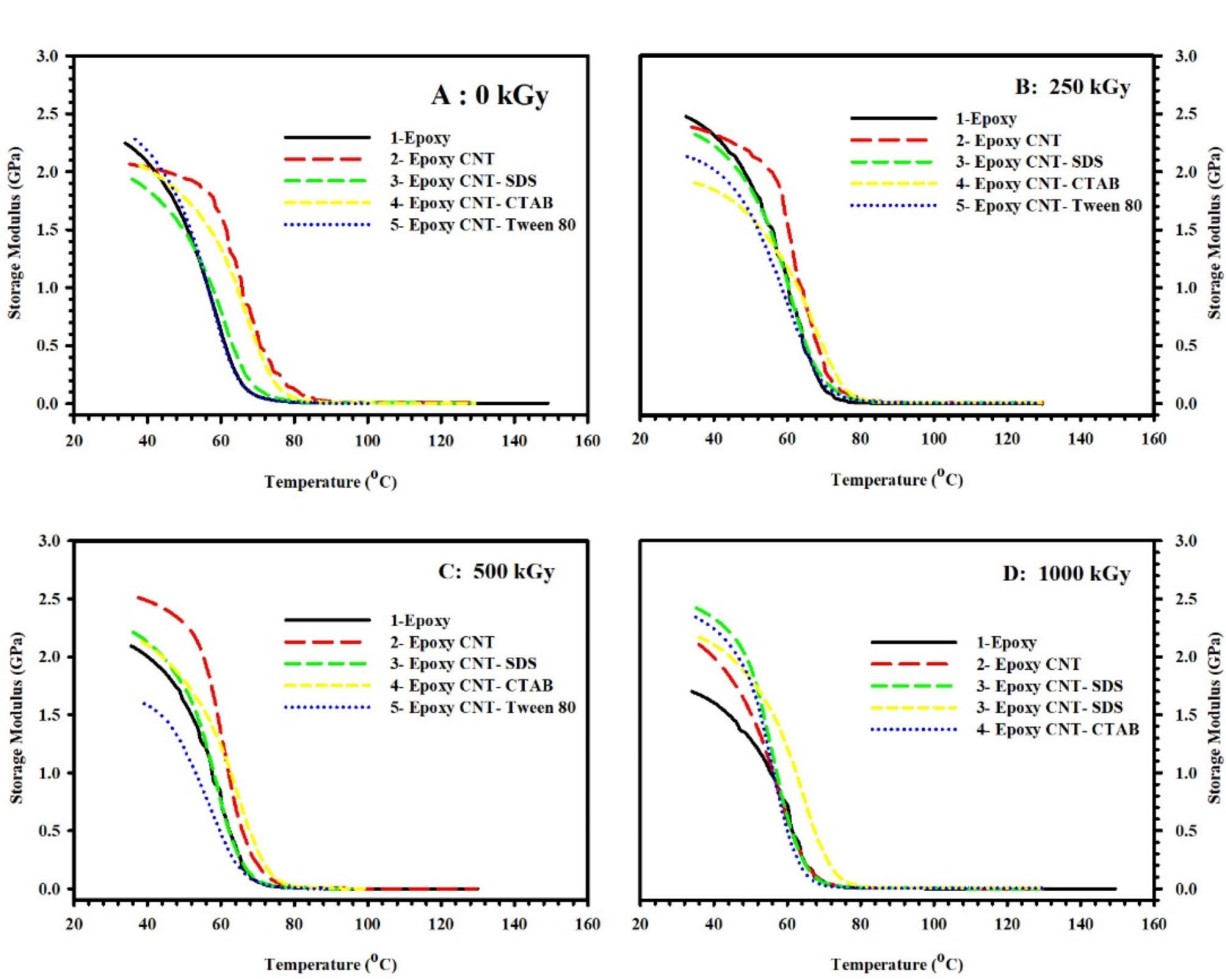
Storage modulus (E´) as a function of temperature (35–100 °C) and fixed frequency 1 Hz for **(A)** 0 kGy, **(B)** 250 kGy, **(C)** 500 kGy, and **(D)** 1000 kGy ɣ-irradiated samples.

Figure 5(A – D) presents E´ for all samples as a function of temperature (35–100 °C) and fixed frequency 1 Hz. The obtained results of Fig. 5(A) for unirradiated samples reveal that epoxy sample loaded with surface modified CNT by Tween 80 had the highest value of E_gr_ (11.01 × 10^3^) among other unirradiated samples. In Fig. 5(B), samples irradiated with 250 kGy dose showed the highest value of E_gr_ for the pristine epoxy sample. On the other hand, results illustrated in Fig. 5(C) revealed the highest value of E_gr_ (43.38 × 10^3^) for epoxy sample loaded with surface modified CNT by Tween 80 among all samples under test. Finally, 1000 kGy dose irradiated samples showed the highest value of E_gr_ (0.83 × 10^3^) for epoxy sample loaded with surface modified CNT by Tween 80 in Fig. 5(D). It is clear from the results that Tween 80 has a strong effect on the soft/hard segment phase separation in epoxy/CNT composites^48^. The values of E_gr_ for all samples under test are gathered in Table 4.

**Table 4:** E_gr_ values (x10^3^) for all samples under test.

	0 kGy	250 kGy	500 kGy	1000 kGy
Epoxy	0.47	41.77	7.62	0.77
Epoxy CNT	0.22	0.29	2.93	0.69
Epoxy CNT SDS	0.33	0.32	15.23	0.78
Epoxy CNT CTAB	0.35	0.31	11.74	0.52
Epoxy CNT Tween 80	11.01	0.39	43.38	0.83

**Fig. 6:**
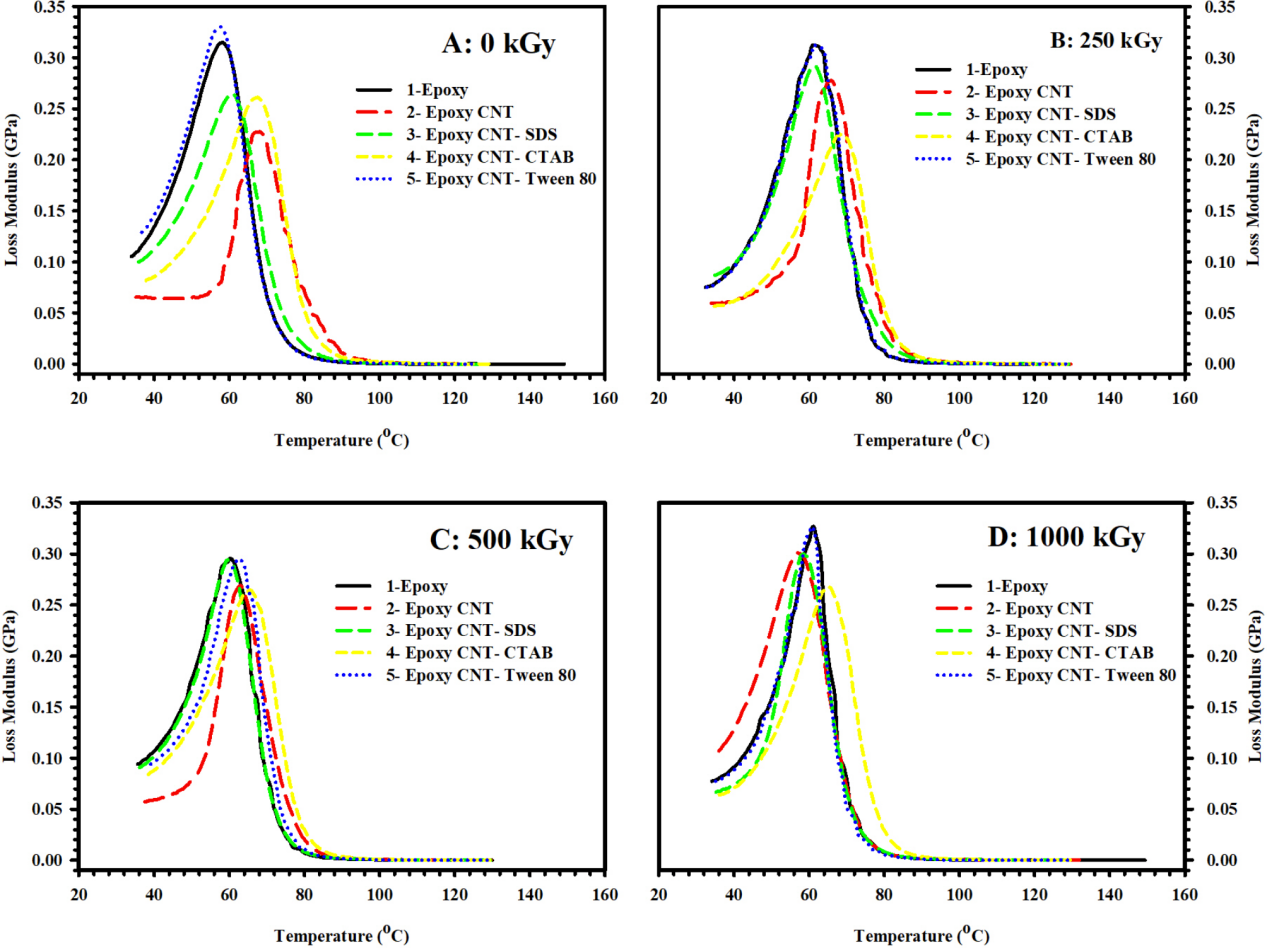
Loss modulus (E˝) as a function of temperature (35–100 °C) and fixed frequency 1 Hz for **(A)** 0 kGy, **(B)** 250 kGy, **(C)** 500 kGy, and **(D)** 1000 kGy ɣ-irradiated samples.

E˝ is plotted as a function of temperature (35–100 °C) and fixed frequency 1 Hz in Fig. 6 for all samples at different radiation doses; 0 kGy (Fig. 6(A)), 250 kGy (Fig. 6(B)), 500 kGy (Fig. 6(C)), and 1000 kGy (Fig. 6(D)). High values of T_g_ are observed for all irradiated and unirradiated epoxy samples loaded with surface modified CNT by CTAB. This infers the strong interaction caused by the CTAB surfactant. On contrary, relatively low T_g_ values are observed for all irradiated and unirradiated epoxy samples loaded with surface modified CNT by Tween 80. This may be attributed to the increased spacing between epoxy chains caused by embedded Tween 80 molecules that create higher free volume and, consequently, higher chain mobility. However, T_g_ values for all samples under test are summarized in Table 5.

**Table 5:** T_g_ values (°C) for all samples under test.

	0 kGy	250 kGy	500 kGy	1000 kGy
Epoxy	58	64	60	59
Epoxy CNT	68	67	63	57
Epoxy CNT SDS	61	61	59	59
Epoxy CNT CTAB	67	68	66	65
Epoxy CNT Tween 80	57	62	58	58

### Crosslinking density

The crosslinking density for all samples is calculated in the rubbery state according to the elasticity theory using the Eqs. 4^2,49^:

$$N=\frac{E^\prime}{3RT}$$

where N is the crosslinking density, E´ is the rubbery region’s storage modulus, while R (8.31 J⋅K^−1^⋅mol^−1^) is the gas constant, and T is the absolute temperature of (T_g_ + 50) K.

The data in Table 6 summarizes the impact of surfactants and irradiation doses on the crosslinking density of all samples. It is obvious that surfactant-free epoxy/CNT sample has higher crosslinking density value than neat epoxy sample. This is attributed to the fact that CNTs can act as catalysts during the curing process of the epoxy resin. They can accelerate the formation of branched chains and crosslinked networks by providing additional reactive sites. Moreover, the high aspect ratio and large surface area of CNTs can contribute to mechanical interlocking within the epoxy matrix. This interlocking can restrict the mobility of polymer chains, leading to a denser crosslinked network^50,51^. However, the role of different surfactants in enhancing the interfacial adhesion between CNTs and epoxy matrix, as well as improving their catalytic activity, is dissimilar^27^. SDS shows a positive impact on the crosslinking density of epoxy/CNT nanocomposites, in contrast to CTAB and Tween 80.

The unmodified epoxy sample exhibits a substantial decline in crosslinking density with increasing irradiation dose, indicating network degradation, particularly at higher doses. In contrast, the inclusion of carbon nanotubes (CNTs) in the epoxy matrix helps maintain a relatively stable crosslinking density at lower doses, though this stability diminishes at higher irradiation levels. The addition of SDS surfactant provides additional stabilization, though the crosslinking density still decreases at elevated radiation doses. CTAB surfactant demonstrates a more pronounced effect, significantly enhancing crosslinking density, particularly at 250 kGy, suggesting a strong interaction with the epoxy matrix that contributes to a more robust network capable of partially withstanding irradiation. Notably, the composite with Tween 80 initially displays the lowest crosslinking density, yet it significantly increases upon irradiation, peaking at 250 kGy, before declining at higher doses, indicating that Tween 80 facilitates network formation under moderate irradiation but may not protect against high radiation doses.

**Table 6:** Crosslinking density (N) values (x10^4^ mol/cm^3^) for all samples under test.

	0 kGy	250 kGy	500 kGy	1000 kGy
Epoxy	4.83	0.86	0.60	0.18
Epoxy CNT	6.97	7.31	0.26	2.40
Epoxy CNT SDS	6.38	6.76	0.86	1.86
Epoxy CNT CTAB	1.55	5.40	0.90	3.63
Epoxy CNT Tween 80	0.64	4.97	1.01	2.87

### Shape memory behavior analysis

**Fig. 7:**
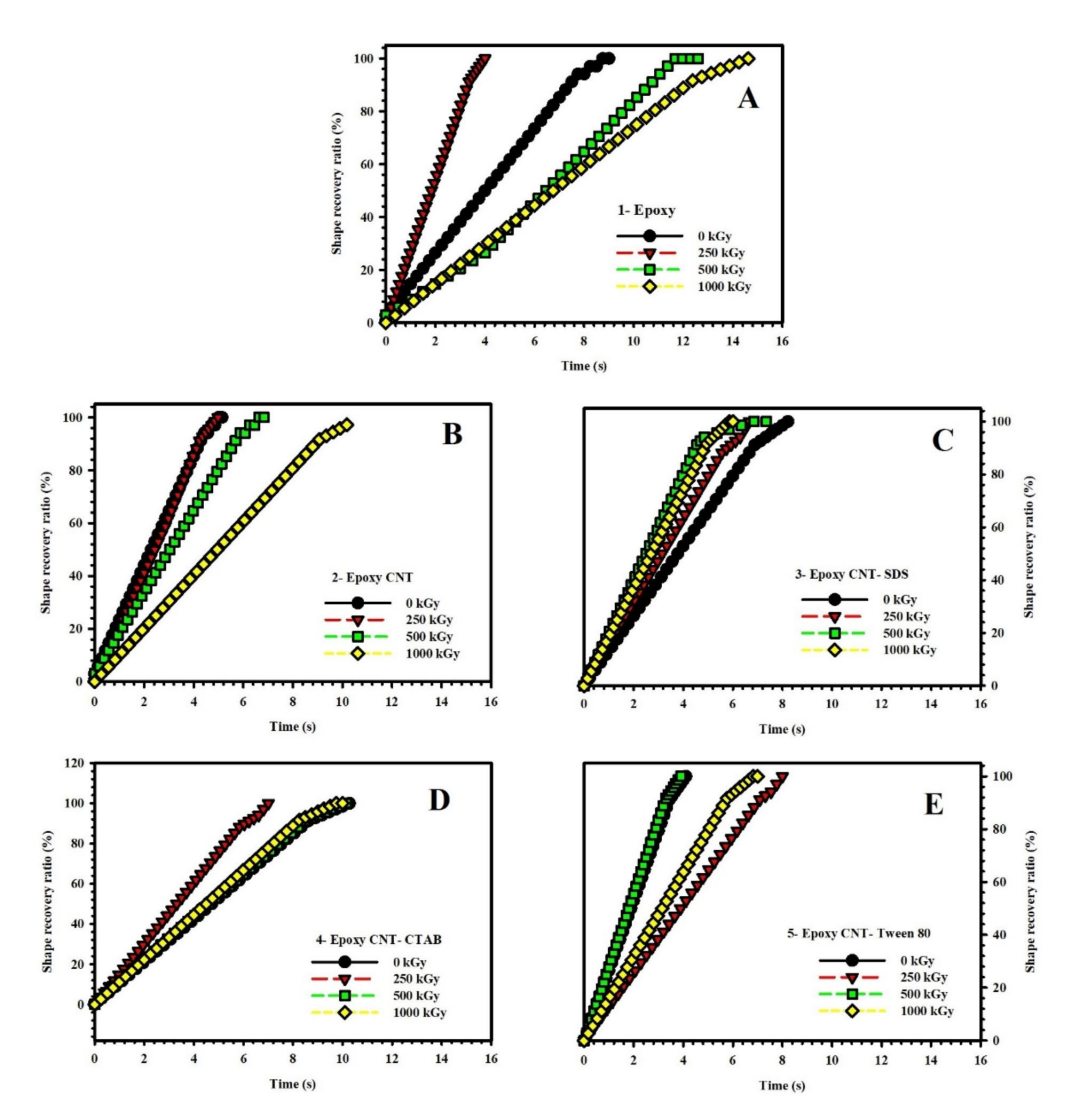
Shape recovery ratio of **(A)** neat epoxy, **(B)** epoxy-CNT, **(C)** Epoxy-CNT-SDS, **(D)** Epoxy-CNT-CTAB, and **(E)** Epoxy-CNT-Tween 80 nanocomposites under different gamma irradiation doses.

**Fig. 8:**
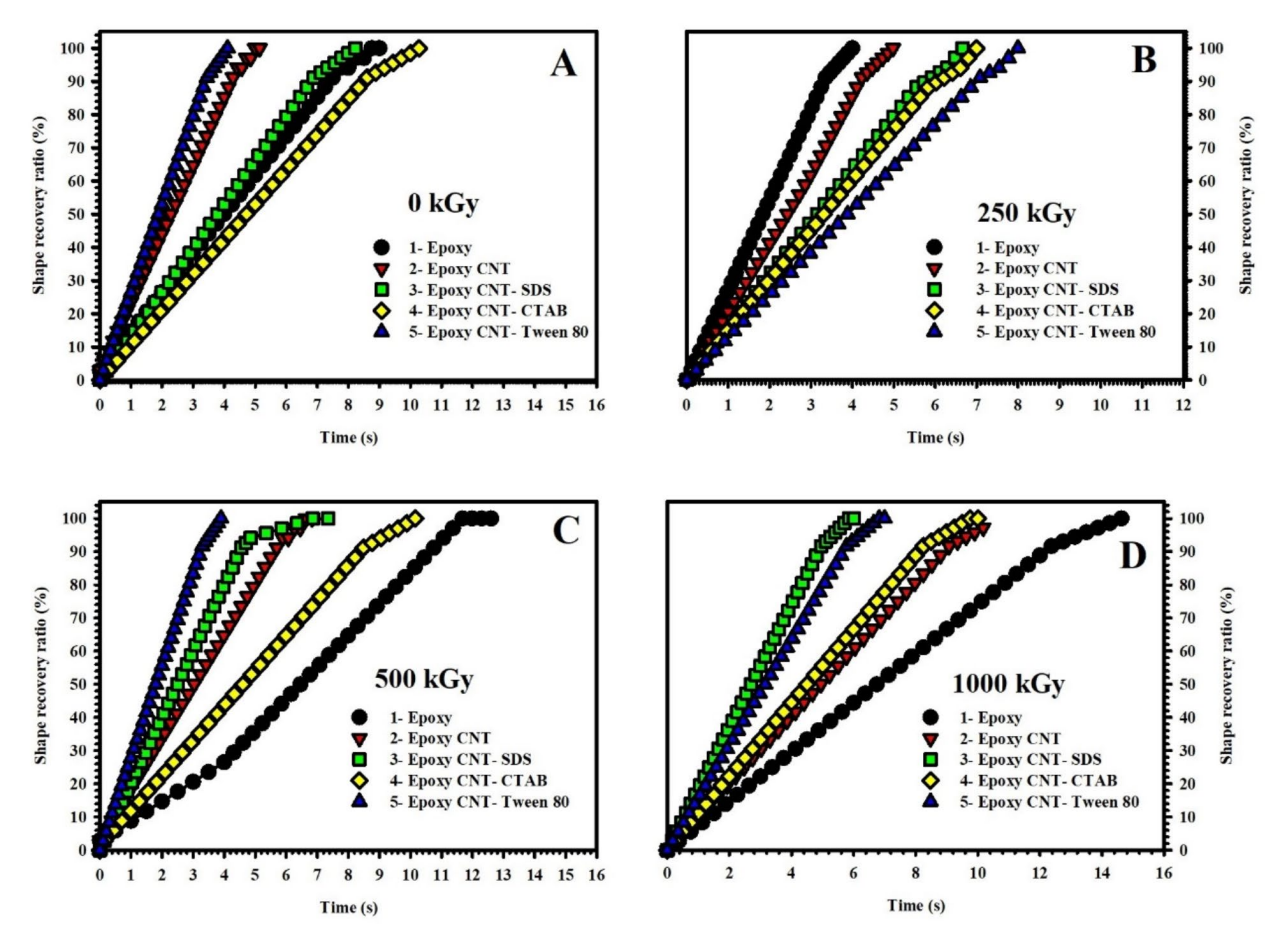
Shape recovery ratio for **(A)** 0 kGy, **(B)** 250 kGy, **(C)** 500 kGy, and **(D)** 1000 kGy ɣ-irradiated samples.

This section presents the outcomes of investigating the shape recovery ratio of epoxy nanocomposites under the influence of gamma irradiation and various surfactants. The shape recovery ratio of nanocomposites examined in this study is represented in five subfigures include; Epoxy (Fig. 7(A)), Epoxy-CNT (Fig. 7(B)), Epoxy-CNT-SDS (Fig. 7(C)), Epoxy-CNT-CTAB (Fig. 7(D)), and Epoxy-CNT-Tween 80 (Fig. 7(E)). Meanwhile, Fig. 8 is plotted to illustrate the effect of radiation doses and consists of four subfigures; 0 kGy (Fig. 8(A)), 250 kGy (Fig. 8(B)), 500 kGy (Fig. 8(C)), and 1000 kGy (Fig. 8(D)). The x-axis of the figures represents the time required for shape recovery in seconds, while the y-axis represents the shape recovery ratio expressed as a percentage.

Upon analyzing the figures, several conclusions can be drawn: 1: Epoxy: The shape recovery ratio time for the Epoxy nanocomposite increases with higher irradiation doses, except for a slight decrease at the 250 kGy dose. At each irradiation dose, the shape recovery ratio time for Epoxy is higher compared to the other nanocomposites. 2: Epoxy/CNT: The shape recovery ratio time for the Epoxy/CNT nanocomposite remains relatively constant across different irradiation doses, indicating that the presence of carbon nanotubes stabilizes the shape recovery behavior. 3: Epoxy/CNT-SDS: The shape recovery ratio time for the Epoxy/CNT-SDS nanocomposite varies with different irradiation doses. At the 0 kGy dose, the shape recovery ratio time is the highest, suggesting that the addition of Sodium Dodecyl Sulfate (SDS) surfactant enhances shape recovery behavior without irradiation. However, at the 1000 kGy dose, the shape recovery ratio time decreases, indicating potential degradation of shape memory properties at high irradiation doses. 4: Epoxy/CNT-CTAB: The shape recovery ratio time for the Epoxy/CNT-CTAB nanocomposite generally increases with higher irradiation doses, indicating a positive effect of the Cetyltrimethylammonium Bromide (CTAB) surfactant on shape recovery behavior. 5: Epoxy/CNT-Tween 80: The shape recovery ratio time for the Epoxy/CNT-Tween 80 nanocomposite fluctuates across different irradiation doses. The highest time is observed at the 250 kGy dose, suggesting a positive impact of the Tween 80 surfactant on shape recovery. However, at the 500 kGy dose, the shape recovery ratio time is the lowest, indicating potential degradation of shape memory properties. The relationship between the irradiation doses and the five surfactant types in epoxy nanocomposites is intricate. The addition of carbon nanotubes and different surfactants can affect the shape recovery behavior, but the specific effects depend on the type of surfactant and the irradiation dose. Further research is necessary to fully comprehend and regulate the interaction between irradiation doses and surfactant types in epoxy nanocomposites.

### Shape fixation rate

**Fig. 9:**
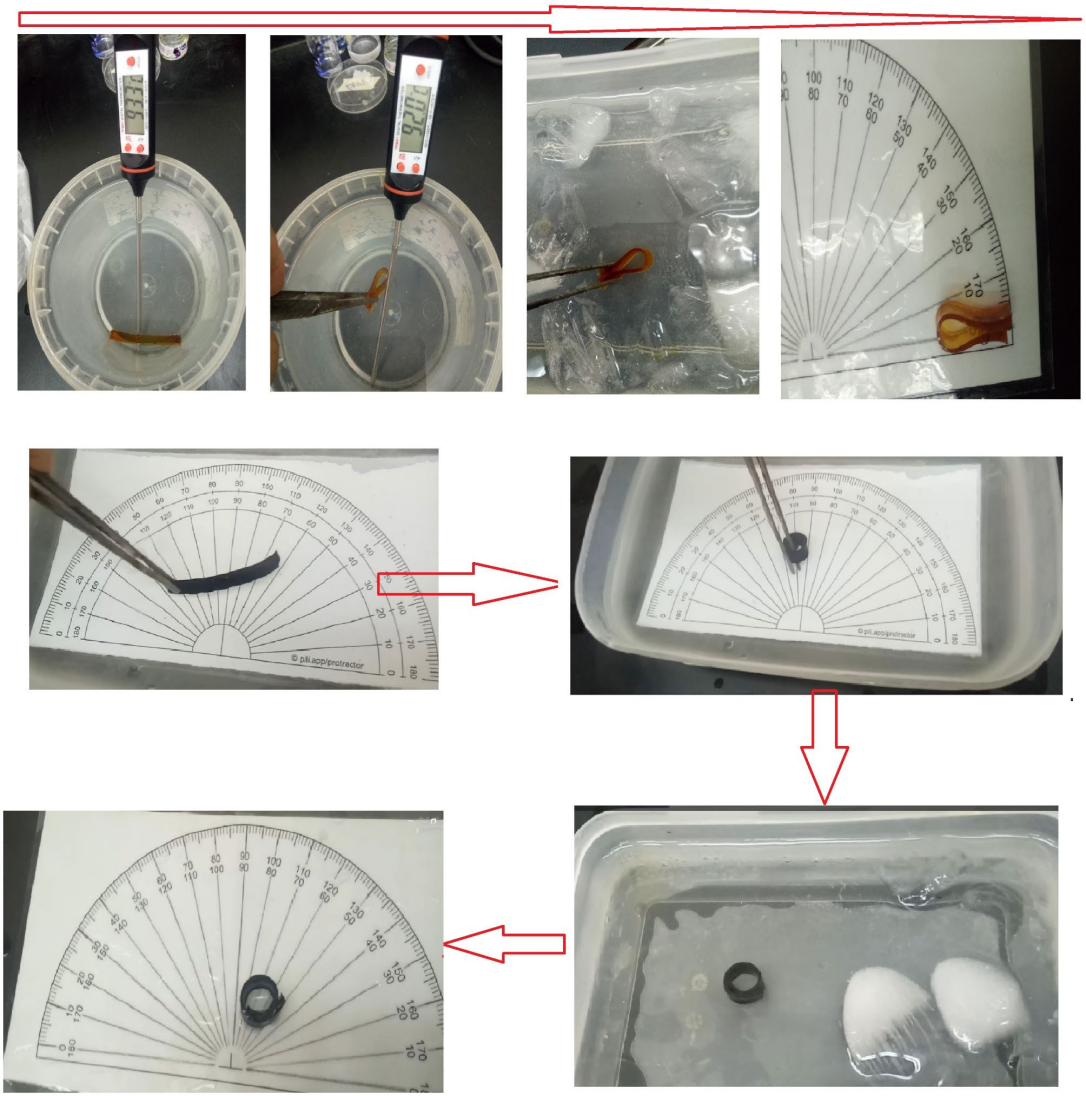
Illustrates sequential images capturing the repetitive processes involved in the shape fixation performance of an epoxy blank and one of the epoxy-CNT nanocomposites.

The fixation rates (also known as the fixity ratio) of selective epoxy, epoxy carbon nanotubes nanocomposites, and different surfactants were evaluated and compared as shown in Fig. 9. Notably, the fixation rate for all the investigated samples remained consistently at 100%. These findings suggest that the incorporation of carbon nanotubes and various surfactants can improve the shape recovery capability of epoxy nanocomposites, with the fixation rate being influenced by the irradiation doses.

## Conclusion

In conclusion, this study delved into the effects of gamma irradiation on epoxy-MWCNT nanocomposites tailored for satellite deployment mechanisms. The meticulous preparation of nanocomposites, coupled with controlled gamma irradiation ranging from 250 to 1000 kGy using the Cobalt-60 facility Industrial Mega Gamma-1 at NCRRT in Egypt, revealed significant insights. Surfactant-enhanced nanocomposites were evaluated, with surface tension measurements conducted in acetone. The surfactants Tween 80 and SDS proved effective in reducing tension to 24.4 mN/m, emphasizing their role in enhancing the dispersion of epoxy-MWCNT composites. Conversely, surfactant-free systems exhibited higher tension at 25.1 mN/m. Cationic surfactant (CTAB) marginally increased tension to 25.4 mN/m but aided in the dispersion of MWCNTs. Nonionic and anionic surfactants exhibited superior dispersing power, aligning well with MWCNTs and contributing to enhanced dispersion. The careful analysis of TGA curves, activation energy profiles, and the effects of Gamma-radiation doses have revealed the intricate relationship between several parameters that affect thermal stability. The strategic application of surfactants, particularly in the epoxy CNT-SDS sample, has been shown to improve thermal stability significantly. Notably, the highest activation energy observed in the Epoxy-CNT-SDS sample indicates a significant hindrance to the degradation reaction, validating the impact of SDS treatment on the crosslinking density of epoxy. Moreover, Gamma irradiation at different doses on epoxy-CNT nanocomposites shows a complex link between radiation exposure and thermal stability. Initially improving decomposition temperature and thermal stability at 250 KGy shows the potential benefits of mild irradiation. Higher doses of 500 and 1000 KGy reduce decomposition temperature and thermal stability, negating this favorable impact. The complex dynamics at play emphasize the necessity of dose management in adjusting these nanocomposites’ thermal characteristics for optimal performance in varied applications. Dynamic Mechanical Analysis (DMA) highlighted radiation-induced changes in viscoelastic properties. Unirradiated epoxy displayed a T_g_ of 58 °C, while 250 kGy irradiation led to enhanced crosslinking with a T_g_ of 64 °C. Higher doses (500 kGy, 1000 kGy) caused marginal T_g_ changes. Surfactant-modified samples showed diverse effects, with Tween 80 emphasizing its role in phase separation. The study underscored radiation’s impact on stiffness and energy dissipation, with shape memory behavior revealing increased recovery time at higher doses, except at 250 kGy. Epoxy-MWCNT demonstrated stable recovery times, suggesting a stabilizing effect of MWCNTs. Fixation rates consistently reached 100%, indicating improved shape recovery influenced by MWCNTs and surfactants. These findings offer valuable insights into optimizing nanocomposites for satellite deployment applications, emphasizing the intricate interplay of gamma irradiation, surfactants, and MWCNTs in shaping the performance of shape memory polymer nano-composites.

### Future prospects

While MWCNTs contribute to consistent shape memory and fixation rates of epoxy/MWCNT SMPs, high irradiation doses increase their recovery time, indicating a potential trade-off between irradiation dose and mechanical performance. Therefore, further studies are necessary to enhance the radiation resistance of epoxy-based nanocomposites, such as investigating alternative surfactants or dispersion methods for MWCNTs as well as investigating alternative nanofillers. Further, long-term exposure tests under simulated space conditions are essential for assessing the durability and performance of epoxy nanocomposites as SMPs for space applications.

On the other hand, repeated cycling between deformed and recovered shapes under different deformation levels can lead to material fatigue, which needs to be addressed over extended periods of time and harsh space conditions. This emphasizes the need for standardized testing and characterization of SMPs and composites for space applications.

Finally, field tests in space-like conditions are necessary to validate laboratory findings and ensure practical applicability in satellite deployment scenarios. In this context, the effect of the space environment, particularly vacuum, on the deployment kinetics and energy consumption of epoxy-based SMPs requires further investigation.

## Data Availability

All data generated or analyzed during this study are included in this published article.
